# Green Synthesis of Er-Doped ZnO Nanoparticles: An Investigation on the Methylene Blue, Eosin, and Ibuprofen Removal by Photodegradation

**DOI:** 10.3390/molecules29020391

**Published:** 2024-01-12

**Authors:** Marília C. R. Silva, Samuel Castro-Lopes, Aimée G. Jerônimo, Ricardo Barbosa, Alexsandro Lins, Pollyana Trigueiro, Bartolomeu C. Viana, Francisca P. Araujo, Josy A. Osajima, Ramón R. Peña-Garcia

**Affiliations:** 1 Programa de Pós-Graduação em Ciência e Engenharia dos Materiais, Universidade Federal do Piauí, Teresina 64049-550, PI, Brazil; marilia.rocha@ufpi.edu.br (M.C.R.S.); bartolomeu@ufpi.edu.br (B.C.V.); araujofp15@gmail.com (F.P.A.); josyosajima@ufpi.edu.br (J.A.O.); 2 Unidade Acadêmica do Cabo de Santo Agostinho, Programa de Pós-Graduação em Engenharia Física, Universidade Federal Rural de Pernambuco, Cabo de Santo Agostinho 52171-900, PE, Brazil; samueljonatas09@gmail.com (S.C.-L.); aimeejeronimo@gmail.com (A.G.J.); ricardojunior1902@gmail.com (R.B.); alexlins1500@gmail.com (A.L.); pollyanatrigueiro@gmail.com (P.T.); 3 Departamento de Física, Universidade Federal de Pernambuco, Recife 50670-901, PE, Brazil

**Keywords:** doped-ZnO, erbium, rare earth doping, *Mangifera indica* gum, sol–gel synthesis, photocatalysis

## Abstract

We present a study on the green synthesis of undoped and Er-doped ZnO compounds using *Mangifera indica* gum (MI). A set of tests were conducted to assess the structure of the material. The tests included X-ray diffraction, Raman, and Fourier-transform infrared spectroscopy. Optical properties were studied using diffuse reflectance and photoluminescence. Morphological and textural investigations were done using SEM images and N_2_ adsorption/desorption. Furthermore, photocatalytic tests were performed with methylene blue (MB), yellow eosin (EY), and the pharmaceutical drug ibuprofen (IBU) under UV irradiation. The study demonstrated that replacing the stabilizing agent with *Mangifera indica* gum is an effective method for obtaining ZnO nanoparticles. Additionally, the energy gap of the nanoparticles exhibits a slight reduction in value. Photoluminescence studies showed the presence of zinc vacancies and other defects in both samples. In the photocatalytic test, the sample containing Er^3+^ exhibited a degradation of 99.7% for methylene blue, 81.2% for yellow eosin, and 52.3% for ibuprofen over 120 min. In the presence of methyl alcohol, the degradation of MB and EY dyes is 16.7% and 55.7%, respectively. This suggests that hydroxyl radicals are responsible for the direct degradation of both dyes. In addition, after the second reuse, the degradation rate for MB was 94.08%, and for EY, it was 82.35%. For the third reuse, the degradation rate for MB was 97.15%, and for EY, it was 17%. These results indicate the significant potential of the new semiconductor in environmental remediation applications from an ecological synthesis.

## 1. Introduction

Zinc oxide (ZnO) is an n-type of semiconductor that has been extensively studied due to its unique properties such as thermal and optical stability, adequate band gap, large excitation binding energy, and affordable cost. These properties make it appropriate for use in various applications such as solar cells, energy storage, optoelectronics and electronic devices [1,2,3,4,5,6,7], and photocatalysis [8,9,10,11]. Additionally, ZnO nanoparticles are non-toxic and biocompatible, making them suitable for various biological applications [12,13,14], which is why they are receiving attention in different research fields.

For photocatalysis research, ZnO is commonly used in combination with other compounds to form heterostructures that exhibit enhanced photocatalytic performance. In this sense, the doping process is a widely employed strategy to modify its properties [15,16,17]. Doping modifies the number of defects within the material, thereby retarding the recombination of electron–hole pairs [18,19]. Notably, ZnO doped with rare earth cations has emerged as an effective method for photocatalytic applications [20,21]. In this context, rare earth ions, in addition to altering the defects concentration within the material, also function as electron traps, leading to increased hydroxyl radical production and consequently an enhancement in the photocatalytic efficiency of ZnO [22,23].

Yu et al. [24] synthesized pure and Er-doped ZnO nanoparticles using the homogeneous precipitation method. The authors revealed that Er-doped ZnO samples significantly enhanced methylene blue (MB) dye degradation. Pascariu et al. [25] obtained pure ZnO nanostructures and doped with 1% of Er, Sm, and La cations using the electrospinning method. The doped samples exhibited superior photocatalytic activity compared to pure ZnO, with the sample doped with Sm showing the highest percentage of Congo red dye removal, approximately 95.8%. Pure and Er-doped ZnO nanoparticles were prepared using the solid-state reaction method [26]. In this study, the authors showed that increasing the concentration of Erbium improved the degradation of methylene blue dye. Furthermore, the sample doped with 0.6 wt% of Er exhibited the highest degradation efficiency. Chemingui et al. [27] synthesized pure and Er-doped ZnO nanoparticles by solid-state reaction. The authors demonstrated that adding Er^3+^ ions increased visible emission, reduced the energy gap, and improved the degradation of the textile dye RR180.

ZnO nanostructures are commonly synthesized through various methods, including precipitation [24], solid-state reaction technique [27], and sol–gel [28]. However, these traditional methods often involve the use of toxic reagents, leading to the generation of toxic products [29]. In this context, the green route is an environmentally friendly approach that minimizes the use of energy and toxic products by utilizing natural and renewable materials during the synthesis process [30,31]. The use of extracts and polysaccharides has been reported to synthesize various ZnO nanostructures [32,33,34,35]. In particular, Vinayagam et al. [34] synthesized ZnO nanoparticles using an extract from *Calliandra haematocephala* leaves. The resulting nanostructures exhibited significant photocatalytic activity, leading to the degradation of up to 88% of methylene blue. Conversely, Araujo et al. [32,33] demonstrated that Gum Arabic or Gum Karaya, when applied for the synthesis of ZnO nanostructures, exhibited excellent photocatalytic properties. Recent reports have indicated the potential of mango leaf extract in the fabrication of nanostructures [36,37]. Panwar et al. [36] employed mango leaf extract to synthesize silver nanoparticles and observed that the resulting nanostructures exhibited good photocatalytic activity. Kumawat et al. [37] synthesized carbon quantum dots using mango leaf extract for application in bioimaging.

Motivated by these findings, this study aimed to use *Mangifera indica* gum as a stabilizer agent for the synthesis of pure and Er-doped ZnO nanoparticles, providing an environmentally adequate approach. Our work focuses on investigating the effect of synthesis parameters and dopants on structural, morphological, and optical properties. Furthermore, for the compound containing Er^3+^ cations, we present a study on methylene blue, yellow eosin, and drug ibuprofen removal by photodegradation. This research is significant because it employs green chemistry in the synthesis of doped nanoparticles for environmental remediation applications aimed at removing toxic organic molecules in an aqueous medium.

## 2. Results and Discussion

### 2.1. Influence of Er^3+^ Insertion on the Structural and Vibrational Properties of ZnO Nanoparticles

**Figure 1:**
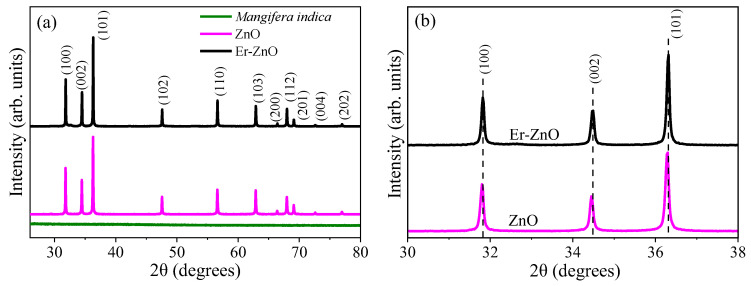
(**a**) XRD patterns for *Mangifera indica* gum, pure, and Er-doped ZnO; (**b**) closer view of the diffraction peaks associated with the (1 0 0), (0 0 2), and (1 0 1) planes.

Figure 1a depicts the XRD patterns for the Zn_1−x_Er_x_O compound. The diffraction peaks corresponding to reflections of the (1 0 0), (0 0 2), (1 0 1), (1 0 2), (1 1 0), (1 0 3), (2 0 0), (1 1 2), (2 0 1), (0 0 4), and (2 0 2) planes, confirm the wurtzite hexagonal structure of ZnO, by the reference code JCPDS No. 36-1451 [38]. In Figure 1a, the XRD pattern of *Mangifera indica* gum (green line) confirms its amorphous nature. Moreover, the utilization of *Mangifera indica* gum and the Er^3+^ ions did not lead to secondary phase formations or the introduction of undesired impurities into the ZnO crystal structure. Figure 1b provides a closer view of the diffraction peaks associated with the (1 0 0), (0 0 2), and (1 0 1) planes. It is noteworthy that the insertion of Er^3+^ ions into the ZnO structure resulted in a shift towards larger angles. This phenomenon can be attributed to the difference in ionic radii between Zn^2+^ (0.74 Å) and Er^3+^ (0.89 Å) [39]. Similar results were reported for rare earth-doped ZnO [40].

The lattice parameters *a* and *c*, the average crystallite size (*D*), and lattice strain (*ε*) were calculated using the equations provided in Table 1. For further information regarding these equations, please refer to References [41,42]. For the ZnO sample, the lattice parameters *a* and *c* values were 3.246(7) Å and 5.202(1) Å, respectively. Meanwhile, for the Er-doped ZnO sample, *a* and *c* were 3.244(6) Å and 5.198(2) Å, respectively. In this instance, the Er^3+^ ions resulted in a decrease in the lattice parameters’ values. This outcome could be attributed to Er^3+^ ions replacing Zn^2+^ ions within the ZnO hexagonal structure [11]. Because Er^3+^ and Zn^2+^ ions have different oxidation states, this substitution may lead to the creation of defects, such as cations vacancies, aligning with the principle of electrical neutrality within the ZnO crystal [43]. These vacancies may be responsible for the reduction in the lattice parameters, a phenomenon previously reported in studies involving rare earth-doped ZnO ceramic [44,45,46].

When comparing the average crystallite size values, it becomes evident that the dopant insertion increased the average crystallite size. In the case of pure ZnO, *D* = 110 nm, whereas for Er-doped ZnO, *D* = 115 nm. This outcome can be attributed to the greater ionic radius of Er^3+^ (0.89 Å) if compared to the Zn^2+^ (0.74 Å). This behavior further supports the notion that Er^3+^ ions replace Zn^2+^ ions within the ZnO structure. Habib et al. [47] demonstrated a similar effect when incorporating Ce^3+^ ions into the ZnO structure, resulting in an enlargement of the average crystallite size. Additionally, Padmavathy et al. [48] found that the ZnO doped with Ag and La cations led to an increase in the average crystallite size due to the larger ionic radii of the dopants. Finally, for pure ZnO and Er-doped ZnO samples, the lattice strain was *ε* = 4.22(2) × 10^−4^ and 2.97(5) × 10^−4^, respectively. In this scenario, since Er^3+^ ions possess an ionic radius larger than that of Zn^2+^ ions, it is plausible that interstitial Zn atoms are affected. Specifically, the Er^3+^ insertion into the substitutional sites may induce the displacement of Zn atoms from their interstitial positions to the grain boundaries, resulting in a reduction in the lattice strain [49].

**Figure 2:**
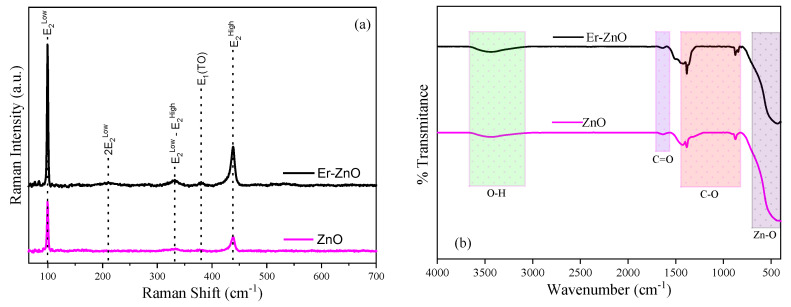
(**a**) Raman spectra and (**b**) FTIR spectra for Zn_1−x_Er_x_O compound.

Raman spectroscopy was employed to investigate how the Er^3+^ ions insertion can impact the vibrational properties of Zn_1−x_Er_x_O compound. In Figure 2a, the Raman spectra of pure and Er-doped ZnO compounds exhibit similarity, with the presence of all the vibrational modes corresponding to the ZnO hexagonal wurtzite structure, confirming the results obtained through XRD analysis. The $E_{2}^{Low}$, 2$E_{2}^{Low}$, and $E_{2}^{High}$ modes were observed at approximately 99 cm^−1^, 211 cm^−1^, and 438 cm^−1^, respectively, for both samples. The $E_{2}^{High}$ mode is associated with the vibration of oxygen atoms, while the $E_{2}^{Low}$ mode is linked to the vibration of Zn atoms [50]. For the pure ZnO sample, the $E_{2}^{High}$ − $E_{2}^{Low}$ mode was observed at approximately 333 cm^−1^, and the dopant insertion caused a shift for 331 cm^−1^. This vibrational mode is associated with the second-order vibration of modes arising to $E_{2}^{Low}$ from $E_{2}^{High}$ the scattering process [51]. Conversely, the $A_{1}(TO)$ mode was observed at approximately 377 cm^−1^ for the ZnO sample and shifted to 380 cm^−1^ for the Er-doped ZnO sample. This peak corresponds to the vibration of O and Zn atoms parallel to the c-axis of the ZnO structure [52]. Nyarige et al. [53] demonstrated that displacements and changes in the intensity of this peak may be linked to the number of defects present in the microstructure of Er-doped ZnO. The displacement on the vibrational modes is connected to structural defects like zinc and oxygen vacancies caused by the insertion of Er^3+^ ions into the ZnO lattice. These findings are consistent with the XRD results, where changes in the structural parameters were detected after the doping process.

The FTIR technique was used to examine the functional groups present in the Zn_1−x_Er_x_O compound. Figure 2b displays the FTIR spectra for pure and Er-doped ZnO nanostructures, within the range of 4000 cm^−1^ to 400 cm^−1^, measured at room temperature. All the vibrational modes observed in pure and Er-doped ZnO samples confirm the ZnO hexagonal wurtzite structure, thereby supporting the results obtained from XRD and Raman spectroscopy [54].

The broad band observed at approximately 3450 cm^−1^ can be attributed to the normal stretching vibration of the O–H function [55]. The peak at approximately 1630 cm^−1^ is linked to C–O stretching vibration, while the region spanning from 1450 cm^−1^ to 820 cm^−1^ corresponds to the stretching of the CH–OH and C–H bonds and ionized carboxylic OH [33,56]. The presence of polysaccharide structure is probably derived from the low calcination temperature that did not eliminate the organic parts. Lastly, the band in the range between 700 cm^−1^ and 400 cm^−1^ is associated with the vibration of the Zn–O bonds [55,57]. The spectra observed for pure and Er-doped ZnO samples exhibit remarkable similarity, mainly because the Er–O vibrations are situated at 569 and 550 cm^−1^, overlapping with the Zn–O vibrations [27]. Therefore, the FTIR results agree with the literature where the presence of carboxylate residues is observed due to the polysaccharide used in the synthesis and confirms the ZnO hexagonal phase formation [58].

### 2.2. Effect of Er^3+^ Cations Inclusion on the Optical Property of ZnO Structure

**Figure 3:**
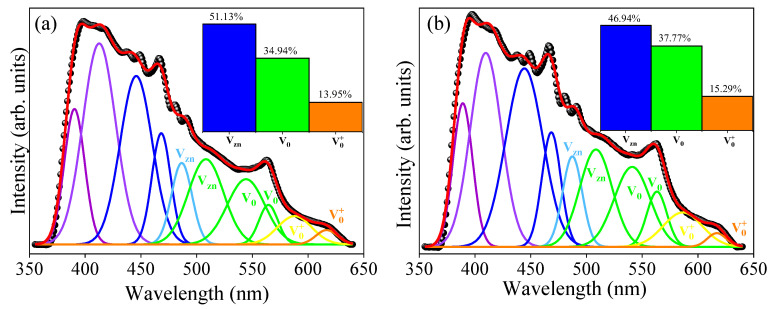
Deconvoluted PL spectra for (**a**) pure ZnO system and (**b**) Er-doped ZnO sample. The insets show the relative percentage of defects in each sample.

Photoluminescence (PL) spectroscopy is a powerful tool for observing the formation of defects in nanomaterials. Typically, the PL spectrum for the ZnO structure exhibits two emission bands. The first band is situated in the UV region and is linked to excitonic recombination between the valence band and the conduction band. The second band is found in the visible region and is attributed to multiple electronic transitions within the material, yielding crucial insights into intrinsic and surface defects, such as oxygen vacancies and zinc vacancies, present in the ZnO crystal structure [59,60]. Figure 3 displays the room temperature PL spectra for the Zn_1−x_Er_x_O compound with wavelength excitation of 340 nm.

The broad emission peak observed in the visible band, featuring multiple electronic transitions, is characteristic of the ZnO hexagonal wurtzite structure and results from the overlap of red, orange, and yellow emissions [61,62]. This finding aligns with results obtained through the XRD, Raman, and FTIR. To gain a deeper understanding of the defects within the material, the PL spectra deconvolutions were performed using a Gaussian function. Three types of defects were identified in the deconvoluted spectra: zinc vacancies ($V_{Zn}$) [470–520 nm], neutral oxygen vacancies ($V_{o}$) [520–570 nm], and singly charged oxygen vacancies ($V_{o}^{+}$) [570–620 nm] [54]. The percentages of these defect types within the Zn_1−x_Er_x_O compound are presented in the bar graph inset in Figure 3a,b.

The pure ZnO sample exhibited a higher concentration of zinc vacancies ($V_{Zn}$ = 51.13%) in comparison to the Er-doped ZnO sample ($V_{Zn}$ = 46.94%). This variation in behavior might be attributed to either the surface of the nanostructures or the grain boundaries, as suggested by Galdámez-Martinez et al. [63]. For the ZnO sample, the percentages of $V_{o}$ and $V_{o}^{+}$ were 34.94% and 13.95%, respectively, while for the Er-doped ZnO sample, they were $V_{o}$ = 37.77% and $V_{o}^{+}$ = 15.29%. As noted, the insertion of Er^3+^ ions into the ZnO structure led to an increase in the number of oxygen vacancies. This phenomenon can be attributed to the substitution of divalent Zn^2+^ ions by trivalent Er^3+^ ions, confirming the outcomes of the structural analysis. A similar outcome was reported by Punia et al. [61] in their study on ZnO nanostructures doped with Gd^3+^. In summary, the analysis of PL spectra allowed us to explore the impact of Er incorporation on the presence of defects within ZnO structures. Modifications in the number of defects hold significant importance for photocatalytic applications [64].

**Figure 4:**
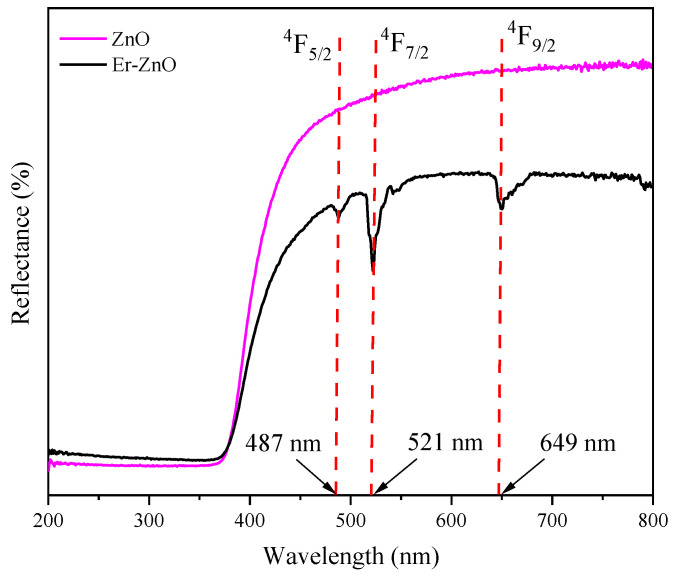
The diffuse reflectance spectra of Zn_1−x_Er_x_O compound.

Diffuse reflectance (DR) measurements were conducted to investigate the impact of Er^3+^ inclusion on the reflectance and bandgap of the Zn_1−x_Er_x_O compound. Figure 4 illustrates the DR spectra, measured at room temperature within a wavelength range of 200–800 nm, for pure and Er-doped ZnO. In both samples, the reflectance starts to exhibit an increase at approximately 375 nm, displaying a pronounced reflective characteristic beyond 450 nm. This behavior signifies that within this wavelength range, photons lack the requisite energy to interact with the electrons or atoms of the material, resulting in a robust reflective capacity [65]. The Er-doped ZnO sample displays a reduction in the reflection band and a redshift. This behavior may be linked to the creation of faulty energy levels within the energy bands around the Fermi level or oxygen deficiency [66]. A similar phenomenon has been reported for ZnO doped with rare earth ions [67,68]. Moreover, the Er-doped ZnO sample exhibits bands at approximately 487 nm, 521 nm, and 649 nm, indicating transitions between the excited levels (4F5/2), (4F7/2), and (4F9/2), and the fundamental level (4I15/2) of Er^3+^ [55,69]. The results obtained through DR provide further evidence of the replacement of Zn^2+^ ions by Er^3+^ ions in the ZnO structure, corroborating the findings obtained from the XRD, Raman, FTIR, and PL measurements.

**Figure 5:**
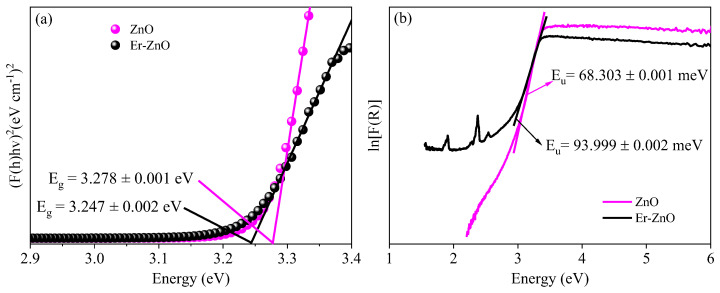
(**a**) Tauc’s plot to estimate the $E_{g}$ energy of the samples and (**b**) Urbach energy for pure and Er-doped ZnO samples.

The optical band gap ($E_{g}$) was estimated using the Kubelka-Munk model [70] and the Tauc equation [71]. Figure 5a presents Tauc’s plot for pure and Er-doped ZnO samples. The band gap value for pure sample is $E_{g}$ = 3.278 ± 0.001 eV, while for sample Er-doped ZnO sample, it is $E_{g}$ = 3.247 ± 0.002 eV. The band gap for the ZnO synthesized sample ($E_{g}$ = 3.278 ± 0.001 eV), is smaller than that of bulk ZnO ($E_{g}$ = 3.37 eV) [72]. However, the value obtained is in good agreement with nanostructures synthesized by different methods, such as co-precipitation ($E_{g}$ = 3.26 eV) [73], aerosol-assisted CVD ($E_{g}$ = 3.24 eV) [74], hydrothermal ($E_{g}$ = 3.20 eV) [75], and sol–gel ($E_{g}$ = 3.26 eV) [50].

Comparing the Eg values of the pure sample with the Er-doped ZnO sample, it is evident that the Er^3+^ insertion promotes a reduction in the optical band gap. This variation is primarily associated with the *sp*-*d* exchange interaction between the *d* electrons located in the 4*f* orbital of the Er^3+^ ions, which replace the Zn^2+^ ions in the ZnO structure [76]. Toma et al. [77] demonstrated that ZnO doped with different rare earth ions (Nd, Gd, Er) led to a reduction in the optical gap due to charge transfer between the conduction band of ZnO and the electrons (4*f* or 5*d*) of the rare earth ions. Another possible reason for band gap reduction could be the formation of intermediate energy levels just below the conduction band [62]. In our case, the higher oxygen vacancy concentration present for the Er-doped ZnO sample may be influencing the reduction in the bandgap value. Costa-Silva et al. [66] demonstrated that the dopant’s inclusion into the ZnO structure results in an imbalance of charges, leading to an increase in defect numbers, such as oxygen vacancies and interstitials zinc, which act as electronic trap centers.

To comprehensively investigate the impact of Er^3+^ insertion on the electronic quality of the Zn_1−x_Er_x_O compound, we evaluated the Urbach energy ($E_{u}$) [78]. Figure 5b illustrates the Urbach energy values for pure and Er-doped ZnO samples. For ZnO sample, $E_{u}$ = 68.303 ± 0.001 meV, while for Er-doped ZnO sample, $E_{u}$ = 93.999 ± 0.002 meV. The dopant inclusion has led to an increase in the Urbach energy value. This effect could be attributed to the higher oxygen vacancy defect concentration observed for the Er-doped ZnO sample, which induces structural disorder and creates localized defect states within the band gap, resulting in a redshift and an increase in the $E_{u}$ value [66]. Furthermore, the electronic states stemming from Er doping will contribute to the preexisting defect states, increasing the Urbach energy [79,80].

### 2.3. Morphological and Textural Changes Induced by the Er^3+^ Ions Inclusion in the ZnO Structure

**Figure 6:**
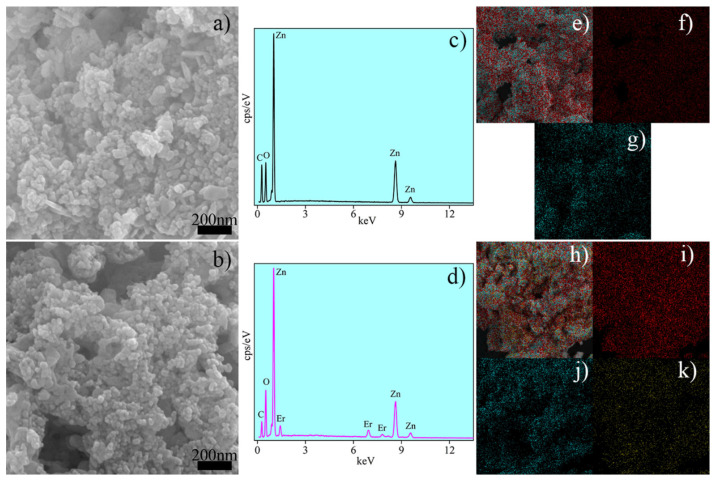
SEM images (**a**,**b**), EDS spectrum (**c**,**d**), and elemental maps (**e**–**k**) of the Zn_1−x_Er_x_O compound.

The morphology of the Zn_1−x_Er_x_O compound was examined using SEM. Figure 6a,b depict SEM images for pure and Er-doped ZnO samples, respectively. Both samples exhibit a similar morphology characterized by the presence of particle agglomerates with varying sizes and shapes. This result suggests that agglomerate formation may result from the nucleation and growth of secondary particles originating from the agglomeration of larger primary particles [81]. These findings are consistent with prior research on ZnO doped with rare earth elements, as reported by other authors [82,83,84,85].

The chemical composition of undoped and Er-doped ZnO samples was examined through energy dispersive spectroscopy (EDS) analysis. Figure 6c,d display the EDS spectra for pure and Er-doped ZnO samples, respectively. In both samples, peaks corresponding to the elements Zn and O were detected, confirming the findings obtained via XRD, Raman, and FTIR. Additionally, the EDS spectrum for the Er-doped ZnO sample (Figure 6d) exhibits signals associated with Er, suggesting the Er^3+^ inclusion into the ZnO structure.

EDS mapping was also conducted to validate the presence and distribution of elements in the ZnO nanoparticles. The EDS maps for the pure ZnO sample are illustrated in Figure 6e–g, whereas the Er-doped ZnO sample is presented in Figure 6h–k. The red and cyan dots, which represent oxygen and zinc, respectively, were detected in both samples. In Figure 6k, the yellow dots represent the Er element, providing straightforward evidence of Er^3+^ cations insertion into the ZnO crystal structure. In summary, EDS analysis confirms that pure and Er-doped ZnO nanoparticles were successfully synthesized using an eco-friendly synthesis method.

**Figure 7:**
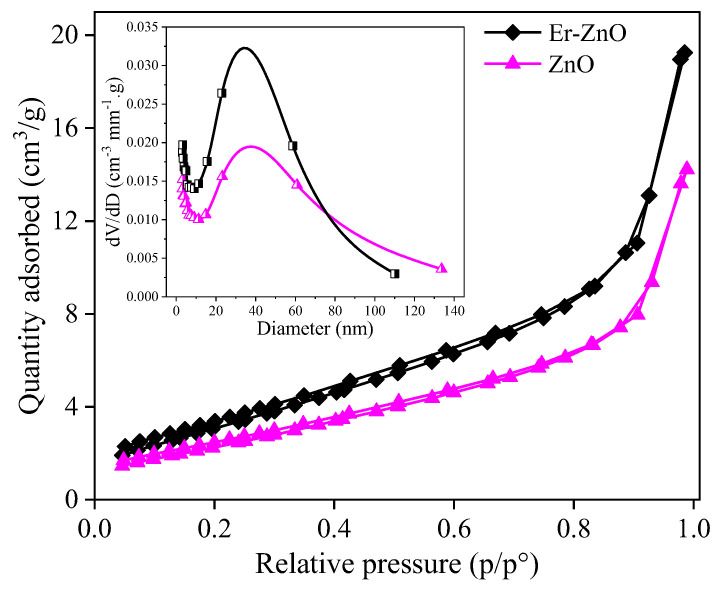
N_2_ adsorption/desorption isotherms at 77 K. The insert presents the pore distribution for pure and Er-doped ZnO nanoparticles.

Figure 7 shows the N_2_ adsorption/desorption results for the Zn_1−x_Er_x_O compound. To determine the textural properties of pure and Er-doped ZnO samples, the specific surface area was calculated using the equation developed by Brunauer, Emmett, and Teller (BET). Additionally, the Barrett, Joyner, and Halenda (BJH) method was used to obtain the average diameter and volume of pores in the adsorption and desorption stages. The shape of the isotherm changes at high relative pressures, and the materials exhibit type IV isotherms, which are characteristic of mesoporous materials. In addition, the H3 hysteresis loops are typical of aggregated materials with no uniform shape and size, as confirmed by SEM images. The pore distribution curves show that the materials have large pores in the mesopore ranges [86,87,88].

Table 2 summarizes the textural properties, where it is observed that there is an increase in the surface area after the dopant cations insertion. These results are favorable for the photocatalytic application of materials because larger surface areas promote the photocatalytic processes.

### 2.4. Photocatalytic Properties of the Zn_0.97_Er_0.03_O Compound

#### 2.4.1. Degradation/Discoloration Tests

**Figure 8:**
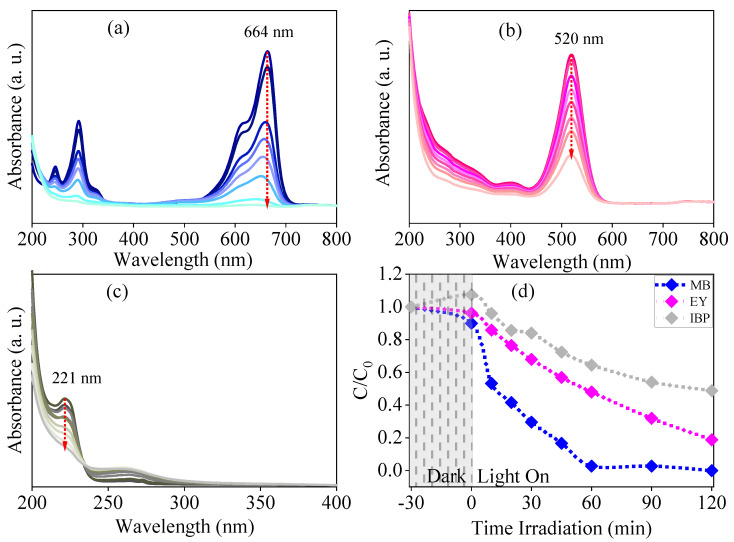
Absorbance spectra of (**a**) methylene blue, (**b**) yellow eosin, and (**c**) ibuprofen. (**d**) Kinetics of photodegradation for MB, EY, and IBU using the Zn_0.97_Er_0.03_O compound under UV source and for the irradiation time *t* = 120 min.

Photocatalytic experiments were conducted to investigate the effectiveness of Er-doped ZnO nanoparticles in breaking down methylene blue and eosin yellow dyes, as well as the ibuprofen drug in aqueous solution under UV light exposure. The maximum absorption band for reference was found to be 664 nm, 520 nm, and 222 nm for MB, EY, and IBU, respectively. Figure 8 shows the removal efficiency, which was determined by plotting the C/C_0_ ratio against the irradiation time. During the photocatalysis test, the pollutant solution was irradiated in the presence of the catalysts. In addition, it was attested that after UV irradiation, the discoloration of MB was 99.77%, for EY was 81.23%, and for IBU, the removal rate was 52.3% after 120 min. These findings indicate the material has excellent photocatalytic activity in removing dyes and drugs from the solution. During the synthesis of the compound, structural changes occurred, as identified by XRD, FTIR, Raman, and PL characterizations… [42,89,90]. These changes have altered the properties of the material, making it more efficient in photocatalytic action [11,40,54,66,81]. This improvement may have been caused by the dopant insertion or by the synthesis with the replacement of the stabilizing agent by gum.

To explain the kinetics of the photodegradation process in heterogeneous photocatalysts, the Langmuir–Hinshelwood model is used. This model describes how the reaction rate is affected by the initial concentration of the reactants [91,92,93]. Once the system reaches adsorption/desorption equilibrium, the reaction constant can be calculated using the following Equation (1):

(1) $$\ln\frac{C}{C_{0}}=K_{app}\times t$$

where *K_app_* is the apparent rate constant [91,94] obtained by the linear slope of the ln C/C_0_ versus irradiation time (*t*) graph. A higher *K*_app_ value indicates a greater efficiency in photodegradation. For example, the degradation rate for MB, EY, and IBU corresponds to 0.0305 min^−1^, 0.0132 min^−1^, and 0.0057 min^−1^, respectively. The values suggest that the Er-doped ZnO nanoparticles are a better photocatalyst for MB than for EY and IBU molecules. In addition, the results indicate that the Er-doped ZnO-based photocatalyst promotes charge separation, improving photocatalytic efficiency.

The photocatalytic efficiency of semiconductor materials depends on various factors such as particle size, morphology, surface area, crystalline structure, and the presence of defects [16,95,96,97]. Apart from these factors, the recombination rate of photogenerated charges can impact the photocatalytic process. This is where dopant and co-dopant come in as alternatives to introduce structural modifications and create defects in the crystal lattice of materials to increase the formation of active species and delay the recombination of electron/hole pairs [17,21,98,99,100,101]. Table 3 presents a comparison of the photocatalytic efficiency of doped ZnO-based semiconductors used in photocatalytic studies of dyes and drugs, drawing on previous research studies.

#### 2.4.2. Scavengers and Recycling Tests

**Figure 9:**
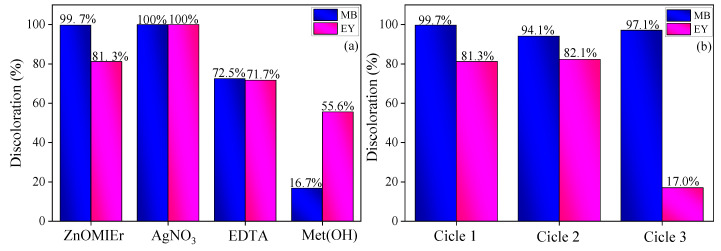
(**a**) Scavengers tests and (**b**) reuse of Er-ZnO in three cycles in the methylene blue and yellow eosin under UV light.

Several tests were conducted to determine the primary agent responsible for Er-ZnO photocatalysis. Methyl alcohol, EDTA, and AgNO_3_ were added to the aqueous dye solution to inhibit the action of specific agents. Methyl alcohol functions as a hydroxyl radical inhibitor, EDTA serves as a hole inhibitor, and silver nitrate acts as an electron inhibitor. In Figure 9a, it was observed that the photocatalysis, in the AgNO_3_ presence, resulted in the complete degradation of MB and EY dyes during the photocatalytic process. This is due to the interaction of silver nanoparticles that undergo photoreduction and adsorb onto the semiconductor surface. They act as electron acceptors and favor the photocatalytic process [109,110]. The EDTA addition in the dye solution resulted in a degradation of 72.5% and 71.7% for MB and EY, respectively. This suggests that holes partially participate in the oxidative decomposition of dyes because they are responsible for the formation of hydroxyl radicals [111]. In the presence of methyl alcohol, the degradation of MB and EY dyes is 16.7% and 55.7%, respectively. Based on these results, it can be concluded that hydroxyl radicals are the active agents responsible for the direct degradation of both dyes [33,56]. During the process of decolorization of the methylene blue, the aromatic rings break and open due to the attack of •OH species. This leads to the decomposition of the phenothiazine structures of these compounds [112]. In the yellow eosin dye degradation, •OH radicals attack benzenic rings and initiate cleavage of C–O and C–C bonds, leading to the complete fragmentation of organic molecules [113].

For a photocatalyst to be efficient, it must have good reuse performance [114]. Therefore, the reuse capacity of Er-doped ZnO nanoparticles was studied over three consecutive cycles (Figure 9b). The nanoparticles, synthesized by green synthesis, underwent three photocatalytic tests, each lasting for a total of 24 h, which was the time required for the collected material by centrifugation to dry and be ready for use. The same pattern, solution dye concentration, and proportion of photocatalyst concentration per dye solution (0.5 g L^−1^) were used in all three photocatalysis processes under UV radiation for 120 min.

The collected material was measured by mass for reuse, and the proportion of dye solution was calculated to maintain the initial concentration. No extra material was added, and the collected material was not washed. In the second reuse, the degradation rate for MB was 94.08%, and for EY, it was 82.35%. In the third reuse, the degradation rate for MB was 97.15%, and for EY, it was 17%. These results show that the material is highly stable when used for decolorization of methylene blue dye. However, for yellow eosin dye, there is a decrease in the ability to remove the dye observed only in the third reuse cycle. This phenomenon may occur due to the formation and adsorption of by-products from eosin photodegradation onto the catalyst surface, leading to decreased efficiency [115].

#### 2.4.3. Degradation/Discoloration Proposal Mechanism

The photodegradation mechanism of many organic molecules has been well-established in the literature. When a photon of light hits the surface of a semiconductor, it provides energy equal to or greater than the bandgap of the semiconductor. This energy causes an electron from the valence band to move to the conduction band, leaving behind a hole [116]. These processes form electron–hole pairs (e^−^/h^+^), promoting oxidation/reduction reactions on the semiconductor surface and facilitating the breakdown of organic molecules. During the photocatalytic process, the trapped electron (e^−^) is captured by oxygen molecules (O_2_) to generate superoxide anions (•O_2_^−^). Simultaneously, the holes (h^+^) act to produce hydroxyl radicals (•OH) due to the reaction with H_2_O on the surface of the material. The trapped electron can then be directed towards oxygen, which boosts the production of superoxide radical anion, acting as a robust reducing agent and resulting in the generation of hydrogen peroxide (H_2_O_2_). Meanwhile, hydroxyl radicals act as an oxidizing agent [117,118,119,120]. Moreover, the dopant (Er^3+^) serves the purpose of accepting the electron during the reaction, thus preventing its recombination. A proposed mechanism of action for Er-doped ZnO sample for decolorization of methylene blue and yellow eosin can be summarized by the following Equations (2)–(9):

ErZnO + hν→ h^+^ (VB) + e^−^ (CB) (2)

h^+^ + H_2_O(ads) → H^+^ + •OH(ads) (3)

e^−^ + O_2_ → •O_2_^−^ (4)

•O_2_^−^+ H^+^ → •OOH (5)

•OOH + H^+^ + e^−^ → H_2_O_2_ (6)

Er^3+^ + e^−^ →Er^2+^ (7)

Er^2+^ + O_2_ → Er^3+^ + •O_2_^−^ (8)

Pollutants + •OH → degradation/discoloration(9)

Regarding photocatalysis, there are two important factors to keep in mind. The competition between the electron removed from the semiconductor’s surface and the recombination of electron/hole pairs is the first. This can be a significant challenge, but doping can be a useful strategy to reduce charge recombination. This can help promote the formation of radical species that enhance the catalytic potential of the photocatalyst [121]. The surface area is another relevant factor in the degradation mechanism because the semiconductor’s surface area affects the number of active sites available for reactions [86,87,88]. The Er-doped ZnO nanoparticles are nano-sized, which results in a greater surface area than ZnO alone. Additionally, suitable crystal size and bad gap energy can enhance the material’s photocatalytic activity [122]. This may be contributing to the discoloration of the dye solutions.

## 3. Materials and Methods

### 3.1. Materials

Reagents of high purity, including zinc nitrate hexahydrate (Zn(NO_3_)_2_·6(H_2_O)) with a purity of 99%, and erbium nitrate pentahydrate (Er(NO_3_)_3_·5H_2_O)) with a purity of 99.9%, were obtained from the Sigma Aldrich Brazil (São Paulo, Brazil) for the synthesis of the compounds. Additionally, distilled water, sodium hydroxide (NaOH), and ethanol were used as solvents, pH control, and for washing, respectively.

### 3.2. Methods

#### 3.2.1. Preparation of *Mangifera indica* Gum

To extract the gum, 20 g of exudate were ground in a mortar and mixed with 100 mL of water. The mixture was stirred constantly on a magnetic stirrer for 14 h. The resulting milky liquid was then filtered to separate it from the solid sticky part. The supernatant was filtered again to ensure that no unwanted material was present. At the start, the pH of the supernatant was 5, so sodium hydroxide (NaOH) was added to neutralize it to pH 7. Next, 99% absolute ethyl alcohol was added to the supernatant to precipitate the gum. The gum formed slowly and appeared as small flakes. Once all the gum was decanted, the solution was centrifuged to separate the gum from the alcohol. The gum was then washed with alcohol three times and dried in an oven at 60 °C for 24 h to obtain the natural polysaccharides.

#### 3.2.2. Preparation of ZnO-Based Nanoparticles

**Figure 10:**
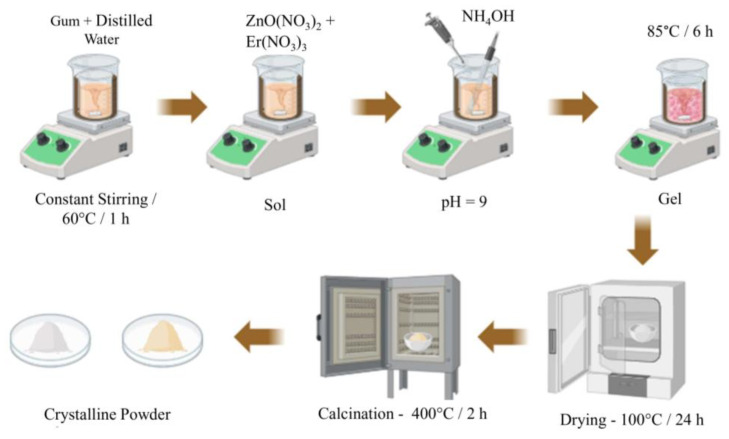
Preparation scheme of pure and ZnO nanoparticles doped with 3% Er^3+^ obtained by sol–gel method.

For the ZnO sample synthesis (Figure 10), 0.5 g of gum was heated in 50 mL of distilled water in a sand bath until the temperature reached 60 °C. Then, 5.9498 g of zinc nitrate hexahydrate was added and stirred continuously for 6 h at an average temperature of 85 °C. The solution obtained had a milky color. Afterward, the solution was dried in an oven at 100 °C for 24 h and then calcined at 400 °C for 2 h.

For the Er-doped ZnO nanoparticles synthesis (Figure 10), 0.5 g of gum was used, which was previously heated in 50 mL of distilled water in a sand bath until reaching a temperature of 60 °C. Then, 5.77 g of zinc nitrate hexahydrate and 0.26601 g of erbium nitrate hexahydrate were added and kept under constant stirring for 6 h at an average temperature of 85 °C. The solution obtained had a pinkish color. The solution went to the oven to dry at a temperature of 100 °C for 24 h and was then calcined at a temperature of 400 °C for 2 h. At the end of the syntheses, two compounds were produced via the sol–gel method at pH 9, the pure ZnO and Zn_1−x_Er_x_O, (x = 0.03), stabilized with 0.5% of natural *Mangifera indica* gum. Each compound was named ZnO for the MI-stabilized pure ZnO compound and Er-ZnO for the MI-stabilized Er-doped ZnO compound.

#### 3.2.3. Characterization Techniques

The samples were characterized by X-ray diffraction (XRD), model D8 Advance from Bruker. Fourier-transform infrared spectroscopy (FTIR) using an Agilent Technology spectrometer, CARY 630 model. Raman spectra were obtained in a mono-grating spectrometer Bruker Senterra. The diffuse reflectance (DR) spectra were measured in a UV-VIS Spectrometer, Shimadzu, UV-2700. The photoluminescence spectra were obtained using a Spectrofluorometer Horiba-JobinYvon Fluorolog-3 with a xenon lamp, 450 W. The micrographics were analyzed in a SEM device, model TESCAN MIRA3; and textural properties were investigated from N_2_ adsorption–desorption employing a Quanta chrome Autosorb-iQ instrument.

#### 3.2.4. Photocatalytic Tests

To investigate the ability of materials to degrade dyes like methylene blue and eosin, as well as the drug ibuprofen, a photochemical reactor made of borosilicate was utilized and placed on top of a magnetic stirrer. The reactor was linked to a thermostatic water bath that kept the solution at a controlled temperature, thus preventing heat from affecting it. A commercial lamp (160 W (emission peak at 350–450 nm)) was placed on top of the reactor (distance of 13 cm from the reactor system), and a Luxmeter was used to measure the light intensity, which was found to be 700 lux. For each test, a concentration of 0.5 g L^−1^ of photocatalyst was added proportionally to 100 mL of MB, YE dye, or IBU drug with a concentration of 10, 10, and 20 mg L^−1^, respectively. The process began with initial agitation for 30 min in dark conditions to reach the adsorption–desorption equilibrium. After this, the solution was irradiated with UV light for 120 min, while 1 mL of the solution was taken out at specific intervals. The samples were centrifuged, and the supernatant was analyzed by using a UV–Vis Spectrometer, Shimadzu, UV-2700. Maximum absorbance was monitored in each solution, where MB = 664 nm, EY = 520 nm, and IBU = 222 nm. Photocatalytic efficiency was calculated using the following expression: Degradation rate (%) = [(C_0_ − C_t_)/C_0_] × 100. Here, C_0_ represents the initial concentration of the solution, and C_t_ is the concentration in the specific time up to 120 min period exposed to UV light irradiation.

The role of reactive species in the degradation of dyes was also evaluated by implementing different inhibitor types, such as methyl alcohol, which inhibits the role of OH; EDTA, which inhibits the action of h^+^, and AgNO_3_, which inhibits the role of e- in heterogeneous photocatalysis. For this, 406 µL of methyl alcohol, 0.0765 g of EDTA, and 0.0086 g of AgNO_3_ were used, along with the same initial amount of photocatalyst and model pollutant. The reuse capacity was also analyzed by performing three cycles. For all reuses, the photocatalysis process was the same as the first photocatalysis, with the amount of substrate balanced about the photocatalyst collected, since it is known that part of the semiconductor is lost during photocatalysis.

## 4. Conclusions

Nanoparticles of pure and Er-doped ZnO were synthesized by the sol–gel method using *Mangifera indica* gum as a stabilizing agent. The calculated parameters demonstrated that the lattice parameters, the average crystallite size, lattice strain, and band gap are influenced by the Er^3+^ dopant ions. Using a Gaussian function, the Urbach energy values and the fit of the photoluminescence (PL) spectra confirmed that the compounds contain high concentrations of V_Zn_, V_o_, and V_o_^+^ defects. The defects affected the behavior of free electrons and created new energy levels, enhancing the photocatalytic potential of the samples. The SEM images confirm the formation of randomly shaped particle clusters, indicating that a natural polysaccharide can contribute to this type of structure. In addition, the samples containing Er (Er-ZnO) showed high efficiency in the photocatalytic test for discoloring MB (99.77%), EY (81.23%), and removing IBU (52.3%) after 120 min. These results indicate that the material has exceptional photocatalytic activity to eliminate dyes and drugs from the solution. Based on the inhibitor test, it can be concluded that hydroxyl radicals are the active agents responsible for the direct degradation of the MB and EY dyes. During the recycling test, it was observed that the Er-doped ZnO sample caused a degradation rate of 94.08% for MB and 82.35% for EY. After the third reuse, the degradation rate for MB was found to be 97.15%, while for EY it was only 17%. These results indicate that the material is highly stable when used for decolorizing methylene blue dye for three consecutive cycles.

## Figures and Tables

**Table 1:** Structural parameters calculated for pure and Er-doped ZnO samples.

Parameters	Equation	ZnO	Er-ZnO
*a* (Å)	$\frac{1}{d^{2}}=\frac{4(h^{2}\;+\;hk\;+\;k^{2})}{3a^{2}}+\frac{l^{2}}{c^{2}}$	3.246(7)	3.244(6)
*c* (Å)	$\frac{1}{d^{2}}=\frac{4(h^{2}\;+\;hk\;+\;k^{2})}{3a^{2}}+\frac{l^{2}}{c^{2}}$	5.202(1)	5.198(2)
*D* (nm)	$\beta cos\theta =\;sin\theta +\frac{K\lambda }{D}$	110	115
*ε* × 10^−4^ (%)	$\beta cos\theta =\;sin\theta +\frac{K\lambda }{D}$	4.22(2)	2.97(5)

**Table 2:** Textural properties of ZnO and Er-doped ZnO ^a^.

Sample	Surface Area (m^2^ g^−1^) ^b^	Pore Volume (m^3^ g^−1^) ^c^
ZnO	9.227	2.032 × 10^−2^
Er-ZnO	12.4	2.756 × 10^−2^

^a^ Nitrogen adsorption/desorption at 77 K. ^b^ Multi point BET method. ^c^ BJH method.

**Table 3:** Comparative studies using doped ZnO-based catalysts.

Catalyst	Catalyst Dosage (g L^−1^)	Target Contaminant	Contaminant Dosage (mg L^−1^)	Radiation Source	Removal (%)	Time of Reaction (min)	Ref.
Dy-ZnO	0.25	Tetracycline Malachite Green Crystal Violet	20	500 W xenon Lamp	74.90, 97.18, 98	120	[102]
Pr-ZnO	1.0	Methyl orange	20	500 W xenon Lamp	>90	90	[103]
La-ZnO	0.5	Methyl blue Ciprofloxacin	10	160 W UV Lamp	91.45 87.6	150	[19]
La-ZnO	0.16	Congo red	60	150 W UV Lamp	97.63	240	[104]
Al-ZnO	0.25	Rhodamine B	4	11 W Hg Lamp	81	120	[105]
Cu-ZnO	0.25	Methylene blue Indigo Carmine Rhodamine B	10	30 W UV Lamp	91.3 92.2 90.1	75	[106]
Cu-ZnO	0.05	Methylene blue	10	Natural sunlight	81	240	[107]
Gd-ZnO	0.33	Methylene blue	10	40 W LED Lamp	93	90	[108]
Er-ZnO	0.5	Methyl blue Eosin yellow Ibuprofen	10 10 20	160 W UV Lamp	99.77 81.23, 52.3	120	This work

## Data Availability

The data presented in this study are available on request from the corresponding author.
